# Large-area graphene-based nanofiltration membranes by shear alignment of discotic nematic liquid crystals of graphene oxide

**DOI:** 10.1038/ncomms10891

**Published:** 2016-03-07

**Authors:** Abozar Akbari, Phillip Sheath, Samuel T. Martin, Dhanraj B. Shinde, Mahdokht Shaibani, Parama Chakraborty Banerjee, Rachel Tkacz, Dibakar Bhattacharyya, Mainak Majumder

**Affiliations:** 1 Department of Mechanical and Aerospace Engineering, Nanoscale Science and Engineering Laboratory (NSEL), Monash University, Clayton, Victoria 3800, Australia; 2 Department of Chemical and Materials Engineering, University of Kentucky, Lexington, Kentucky 40506, USA

## Abstract

Graphene-based membranes demonstrating ultrafast water transport, precise molecular sieving of gas and solvated molecules shows great promise as novel separation platforms; however, scale-up of these membranes to large-areas remains an unresolved problem. Here we demonstrate that the discotic nematic phase of graphene oxide (GO) can be shear aligned to form highly ordered, continuous, thin films of multi-layered GO on a support membrane by an industrially adaptable method to produce large-area membranes (13 × 14 cm^2^) in <5 s. Pressure driven transport data demonstrate high retention (>90%) for charged and uncharged organic probe molecules with a hydrated radius above 5 Å as well as modest (30–40%) retention of monovalent and divalent salts. The highly ordered graphene sheets in the plane of the membrane make organized channels and enhance the permeability (71±5 l m^−2^ hr^−1^ bar^−1^ for 150±15 nm thick membranes).

## Abstract (web summary)

Membranes made from graphene have ultra-fast water transport and precise molecular sieving properties. Here, the authors show how large-area membranes can be manufactured by a rapid and scalable process based on shear alignment of graphene-oxide liquid crystals for unlocking industrial applications.

## Graphical abstract

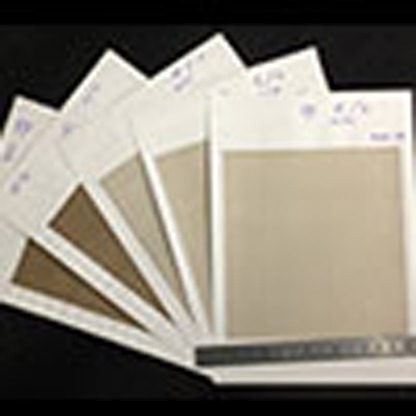

Advances in the design and synthesis of nanofiltration membranes with improved retention, flux and cost-effectiveness will have tremendous impact in several fields such as water treatment, selective chemical separations and drug delivery. Conventional polymeric nanofiltration membranes usually have limited chemical resistance, while ceramic membranes are not cost-efficient. Graphene is a one atom thick two-dimensional honeycomb sp^2^ carbon lattice, which is an exciting multifunctional material and possesses a combination of strong mechanical properties, chemical inertness and extremely large surface area^1^,^2^. Membranes prepared from graphene possess the best of both the worlds: they are chemically inert^3^ like ceramic membranes and can be made into films using graphene/graphene oxide (GO) fluid phase dispersions like polymers. Novel and exciting transport properties of graphene-based membranes such as high permeability and high selectivity for both liquids^2^,^4^,^5^,^6^,^7^ and gases^8^,^9^,^10^,^11^ have recently been reported. While these studies have unlocked potential applications, there is critical need to produce these membranes in large-areas using high throughput manufacturing routes, which may otherwise hinder their impact in membrane technologies. The ideal structure of a filtration membrane has a defect-free, thin, dense separation film that acts as a functional sieve, while the mechanical strength is provided by a porous and more permeable support. To achieve this asymmetric structure, researchers have grown continuous graphene films by chemical vapour deposition and transferred them to substrates followed by etching pores on the film, however, the transfer process limits the scalability of membrane production^6^,^7^. Another method to produce this structure is by restacking GO flakes by filtration of GO dispersions on a backing filter support^2^,^12^,^13^,^14^. However, producing a membrane by this approach requires large volumes of liquid, significant time and arguably has both alignment (of the GO sheets) and scalability issues. Other liquid phase processes such as dip-coating or layer-by-layer assembly similarly have potential issues with rapid productivity^15^. Therefore, a major challenge in this field is to define robust, scalable, liquid film processing approaches to produce large-area graphene-based membranes that will bridge laboratory curiosity to industrial productivity.

Here we introduce a scalable and industrially adaptable method to fabricate large-area graphene-based membranes by shear-induced alignment of liquid crystals of GO. The membranes have large in-plane stacking order of GO sheets and demonstrate outstanding water permeability while being able to sieve small organic molecules with performance metrics superior to well established and commercially available nanofiltration membrane.

## Results

### Producing liquid crystalline phases of GO

**Figure 1:**
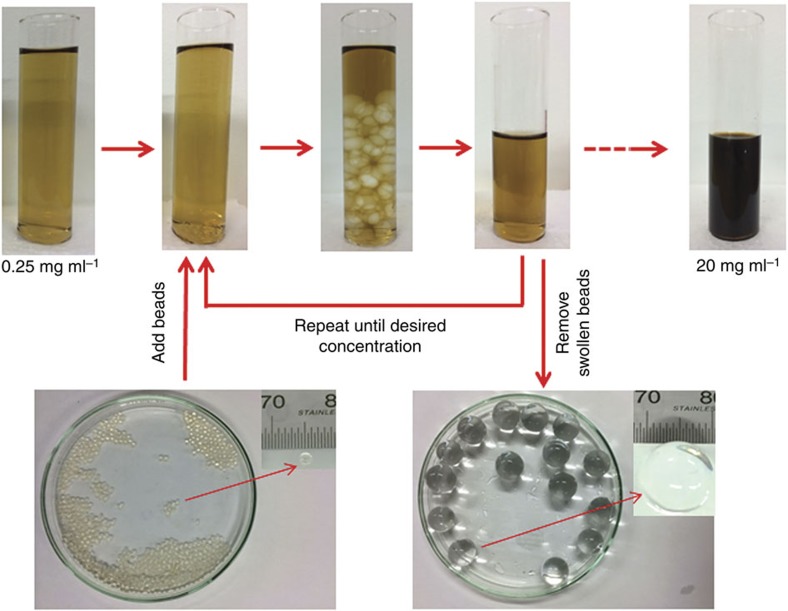
Procedure for concentrating GO. Photographs of stable dispersions of GO which have been concentrated by adding hydrogel beads.

**Figure 2:**
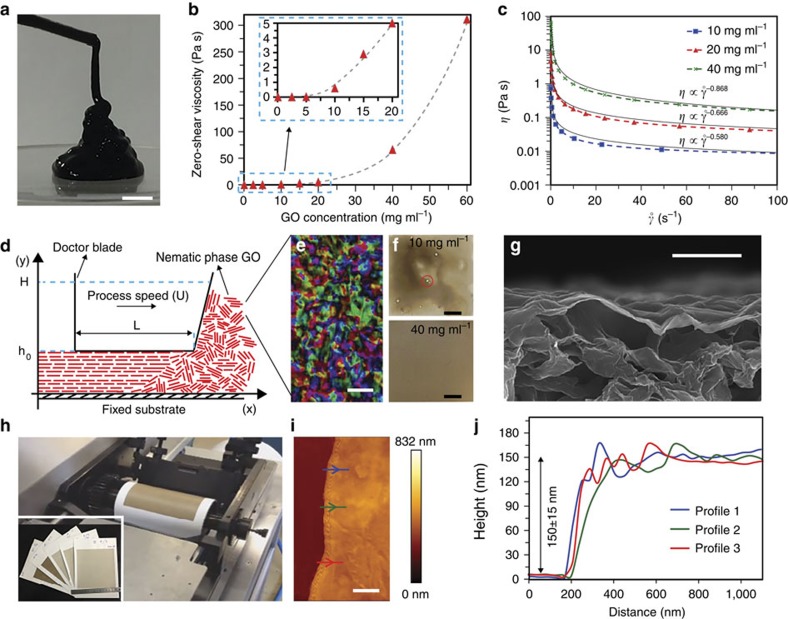
Fabrication of shear-aligned membrane from nematic GO. (**a**) Viscoelastic property of GO (∼40 mg ml^−1^). Scale bar, 1 cm. (**b**) Zero-shear viscosity of the dispersions increases with increasing GO concentration. Dashed line is a polynomial fit. (**c**) Rheology data for three different concentration showing shear-thinning behavior. Solid curves are the fit of the experimental data with a power law model. (**d**) Schematic of shear-alignment processing of a nematic GO to a film; L is the width of blade, h_0_ is the height of the channel, H is the height of the fluid in front of the blade and U is the processing speed. (**e**) Polarized light images of fully nematic GO at 40 mg ml^−1^ (scale bar, 1 μm). (**f**) The red circle in the photograph identfies dewetting spots in the SAMs, which is eliminated when processed from liquid crystalline GO (scale bars, 1 cm). (**g**) An SEM image demonstrates continuity and conformity of SAM over a porous Nylon substrate (scale bar, 1 μm). (**h**) Photograph of the gravure printing machine and (inset) images of 13 × 14 cm^2^ GO membranes with different thicknesses. (**i**,**j**) AFM height map and corresponding height profiles of our membrane (scale bar, 1 μm).

Oxidation and exfoliation of graphite by the well-known Hummers' method or its variations produces graphene nanosheets decorated with oxygenated functional groups also known as GO. The anisotropic GO nanosheets can be dispersed in liquids including water as stable colloidal suspensions with large volume fractions. As the concentration of the anisotropic particles increases, the orientation entropy of the suspensions starts to decrease only to be compensated by increase in the translation entropy leading to colloidal phase transitions from isotropic to nematic liquid crystalline phases—the onset of which has dependence on the thickness to diameter ratio of the disc-like mesogens of GO^16^. Liquid crystallinity defines a state between a crystal and a fluid, within which the constituent sheets become anisotropic but can still flow and respond to macroscopic force-fields such as shear^17^, and this state has been demonstrated in concentrated dispersions of GO^1^,^18^,^19^,^20^. Traditional means to produce concentrated GO dispersions, such as the application of heat^21^ or the use of vacuum equipment^22^, are time consuming and laborious. An innovative method was implemented in this work to quickly produce nematic GO dispersions (Fig. 1). We used superabsorbent polymer hydrogel beads (typically, cross-linked polyacrylate based copolymer), which are strongly hydrophilic. Concentration of a GO dispersion occurs because the hydrogel beads absorb and retain water^23^ without dissolving in water or absorbing GO sheets. This is demonstrated in Raman characterization in Supplementary Fig. 1 (Supplementary Note 1)—the characteristic peaks of GO were not observed within the hydrogel beads swollen in a GO suspension. The time taken to concentrate a GO dispersion depends on the initial concentration, the desired concentration and the mass of beads used. Fig. 2a shows a GO dispersion with a concentration of 40 mg ml^−1^.

### Rheological characterization of GO dispersions

Rheological properties of the GO dispersions are crucial to our fabrication method. We evaluated zero-shear viscosity by measuring the viscosity of the GO dispersions at a shear rate of 0.001 s^−1^ (ref. 24). Fig. 2b demonstrates that the zero-shear viscosity increases with an increase in GO concentration. At low concentrations of GO, water molecules are attached to GO sheets via hydrogen bonds^25^, but similar to water-clay dispersions^26^, an increase in the number of GO sheets results in the assembly of graphene sheets and water molecules to form a three-dimensional network via hydrogen bonding, which decreases the fluidity of the dispersion. Furthermore, the large changes in the viscosity beyond 5 mg ml^−1^ coincides with the onset of liquid crystalline nematic phase^16^. Fig. 2c presents apparent viscosity of the GO dispersion (*η*) as a function of shear rate (˚γ). The non-Newtonian shear-thinning (pseudoplastic) behaviour was observed at different concentrations of the GO dispersion. Decreased viscosity of the GO dispersions with an increase in the shear rates is consistent with previous reports^18^,^27^. One can presume that the nematic phases in the GO dispersion are distributed randomly and do not align at low shear rates, which results in higher viscosity. At high shear rates, the randomly distributed nematic phases align in the direction of shear stress and produces less physical interaction with each other, resulting in decreased viscosity. Fig. 2c shows that the viscosity of the GO dispersions were in good agreement with the power law viscosity model. In the power law model, the exponent for ideal plastic material is −1 and any deviation from this theoretical value shows a loss of plastic behaviour^28^. The exponents decrease from −0.580 to −0.867 by increasing GO concentration from 10 to 40 mg ml^−1^, which affirms the increased plasticity arising from the nematic GO phases.

### Interfacial properties of GO dispersion

Properties of the GO dispersion, typically at the solid–liquid interface, also assume importance in our membrane fabrication process. Several criteria need to be satisfied to ensure accurate measurement of the surface tension and the contact angle^29^,^30^: the droplet has to be symmetric along the central vertical axis, the droplet should be shaped only by gravity and surface tension forces and no other forces such as viscosity should play a role in the motion or inertia of the droplet. Droplets formed by 40 and 60 mg ml^−1^ GO dispersions did not satisfy these criteria due to high viscosity, so the surface tension and contact angle values were estimated by linear extrapolation (Supplementary Tables 1 and 2) for these two cases. The argument for using linear extrapolation for these dispersions is that the surface tension of GO dispersions decreases with increase in GO concentration given that GO possesses surfactant-like properties^31^. The contact angle between the GO dispersion and the Nylon substrate decreases with decreasing surface tension, which is consistent with inverse correlation between contact angle and surface tension in the Young's equations^26^:


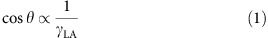


where, *θ* is contact angle between the GO dispersion and a Nylon substrate, and *γ*_LA_ is the interfacial surface tension of the GO dispersions.

### Membrane fabrication

The primary goal of our work is to form large-area GO membranes by taking advantage of the discotic nematic phase of GO by a shear-induced, industrially adaptable liquid thin film process (Fig. 2d) referred to in this article as shear-aligned membrane (SAM). The discotic nematic colloidal phase (Fig. 2e) has a crucial role in enabling membrane formation, which goes beyond the requirements of high solid contents necessary to produce a continuous film. The GO colloidal dispersion used in our studies undergoes an isotropic to nematic phase transition at ∼5 mg ml^−1^, remaining bi-phasic until ∼15 mg ml^−1^, and fully nematic phases are formed at higher concentrations beyond 16 mg ml^−1^ (ref. 16). Typical physical properties of the GO colloidal suspensions representing isotropic, bi-phasic and fully nematic phases are shown in Table 1.

**Figure 3:**
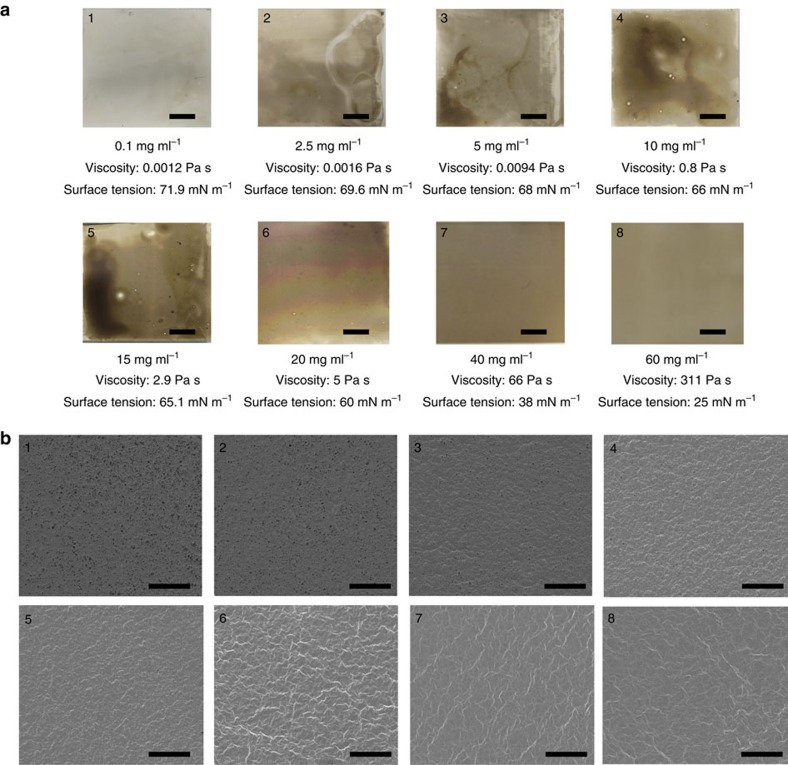
Effect of GO concentration on uniformity and continuity of film. (**a**) Photographs (scale bars are 1 cm) and (**b**) SEM images (scale bars, 50 μm) of top surface of the shear-aligned membranes cast by progressively increasing concentration: (**1**) 0.1 mg ml^−1^, (**2**) 2.5 mg ml^−1^, (**3**) 5 mg ml^−1^, (**4**) 10 mg ml^−1^, (**5**) 15 mg ml^−1^, (**6**) 20 mg ml^−1^, (**7**) 40 mg ml^−1^, (**8**) 60 mg ml^−1^.

The nematically ordered fluid phases of GO have non-Newtonian flow characteristics (Fig. 2b,c), which can be harnessed to produce large-area films by using shear forces as in doctor blading and dip coating^32^,^33^,^34^. Our choice of a rigid blade (known as doctor blade in industrial terminology) as the shear alignment method is dictated by factors including its use in large-scale, continuous, high-speed, liquid thin film processes as the metering, applicator gadget and wide-scale use in preparing polymeric films^35^. The size of a membrane that one can produce is limited only by the size of the shearing apparatus, thus large-area membranes can be produced with relative ease. We also hypothesize that the high shear stress will orientate the graphene sheets of nematic discotic phase^17^,^36^, packing them into a dense, continuous, uniform membrane over a porous support in a rapid single step (Fig. 2d). To investigate this, we initially used a lab-scale doctor blade that spreads the fluid under Couette flow through a thin rectangular channel (Supplementary Fig. 2 and Note 2). The viscosity of the fluid is the dominant material parameter in the imposed shear stress: 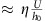, where *τ* is shear stress, *η* is viscosity of the GO dispersion, *U* is process speed and *h*_0_ is the doctor blade gap size (Fig. 2d). GO fluids from 0.1 to 60 mg ml^−1^ were studied with systematic variation of viscosity. Photographs (Figs 2f and 3a) and scanning electron microscopic (SEM) images (Figs 2g and 3b) revealed that the uniformity of the cast film increased with increasing GO concentrations. The films made by 40 and 60 mg ml^−1^ GO suspensions have best uniformity and continuity. To demonstrate the proficiency of our approach in membrane production, we made large-area GO membranes (40 mg ml^−1^) by a gravure printer (Supplementary Movie 1), with thicknesses ranging from ∼65 to ∼360 nm, on porous Nylon substrates. Although several methods using solution chemistry or energetic radiation can be used to chemically reduce the membranes, as a proof-of-concept to stabilize the membranes in aqueous environment, we partially reduced the GO membranes by exposure (∼5 min) to hydrazine vapour^37^,^38^.

### Nanofiltration performance

**Figure 4:**
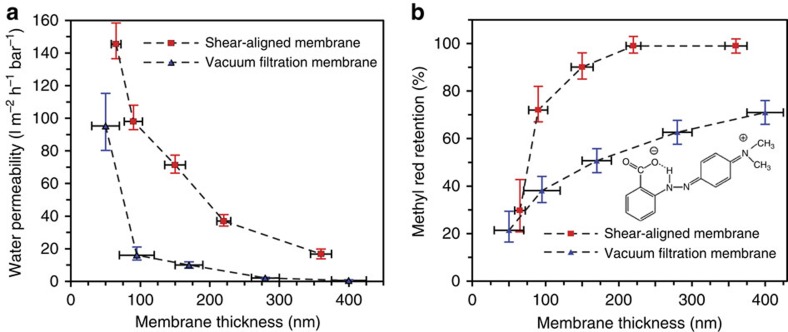
Comparision of SAM and vacuum filtration membrane performance. (**a**) Water permeability versus thickness, and (**b**) Retention of methyl red, an electroneutral probe molecule. Inset of **b** is the structure of the methyl red. Error bars in these figures are from five measurements showing the maximum and minimum values.

**Figure 5:**
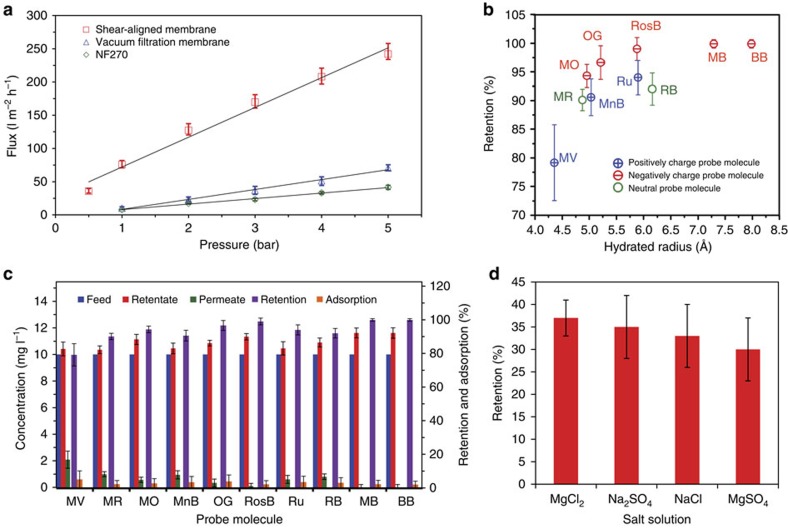
Filtration performance. (**a**) Water flux versus applied pressure for three different membranes: SAM (red) with a thickness of 150±15 nm, vacuum filtration (blue) with a thickness of 170±20 nm, and NF270, a commercial nanofiltration membrane (green). SAM showed a retention of 90±2% for methyl red, while the vacuum filtration membrane and NF270 showed 50±5% and 90±1.5% retention, respectively. (**b**) Retention performance of the 150±15 nm thick shear-aligned membrane, as a function of hydrated radius, for probe molecules with different charges and sizes. (MV is methyl viologen, MR is methyl red, MnB is methylene blue, MO is methyl orange, OG is orange G, Ru is Tris (bipyridine) runthenium (II) chloride, RB is Rhodamine B, RosB is Rose Bengal, MB is methylene blue, BB is brilliant blue. The green, red and blue symbols represent electroneutral, negatively and positively charged probe molecules, respectively. (**c**) Retention details of the membrane for the probe molecules. (**d**) Salt retention by the 150±15 nm thick SAM, for four different salt solutions. Error bars in these figures are from five measurements showing the maximum and minimum values.

Performances of the membranes as a function of membrane thickness were first evaluated by measuring the water permeability (using Reverse Osmosis water, known as RO water) and the retention for Methyl Red (an electroneutral probe molecule at pH∼5.5 (ref. 39); Fig. 4a,b). Membranes with ∼150 nm thickness were found to exhibit the most promising trade-off between flux and retention. Consequently, the SAMs with ∼150 nm thickness was chosen for further characterizations. We have compared performance of the SAM with those prepared using the vacuum filtration technique^2^,^12^,^13^,^14^. Different thicknesses of GO membranes were prepared by changing the volume of the GO solution (10 μg l^−1^) in the vacuum filtration process. These GO membranes were further reduced via hydrazine vapour following the same methodology used for SAM. We compared the water permeability and the retention of methyl red, a probe molecule that is electroneutral at the experimental pH (∼5.5)^39^, for the SAM and the vacuum filtration membranes, with varying membrane thickness, measured here by AFM (Fig. 2i,j). While it is not surprising that the retention is enhanced, the water permeability is also improved as a result of the stacking order in the SAM. Water flux versus pressure measurements for three different varieties of membrane: SAM, vacuum filtered and a commercial membrane (NF270 membrane, Dow Chemical Company, USA) are shown in Fig. 5a. The SAM had a water permeability of 71±5 l m^−2^ hr^−1^ bar^−1^, which is almost seven times better than vacuum filtration membranes (10±2 l m^−2^ hr^−1^ bar^−1^) and almost nine times better than the NF270 membrane while demonstrating comparable or better retention for the electroneutral probe—methyl red (Fig. 4b).

The flux through the membrane increases linearly with increasing applied pressure (Fig. 5a). The modified Hagen-Poiseuille equation for slit-shaped pores^13^ (Flux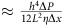) gives an approximate explanation of fluid flow through these multi-layered structures. Using this equation one can estimate the mass flow rate of a Newtonian fluid through porous materials per unit area (m^3^ s^−1^ m^−2^), where *h* (m) ∼0.95 × 10^−9^ is the distance between neighbouring graphene sheets (estimated from X-ray diffraction, Supplementary Fig. 3), Δ*P*=0.5 × 10^5^ Pa is the pressure gradient, *L*=0.9 × 10^−6^ m is the average lateral length of the graphene sheets, *η*=0.001 Pa s is the viscosity of water at 20 °C, and Δ*x*=150 × 10^−9^ m is the thickness of the membrane. Comparison of the experimental results with estimated fluxes from the modified Hagen-Poiseuille equation reveal that the theoretical fluxes are four orders of magnitude smaller than the experimental results. This experimental enhancement is consistent with reports of water transport in nanotubes^40^ and the slit-pores of graphene^2^,^13^.

**Figure 6:**
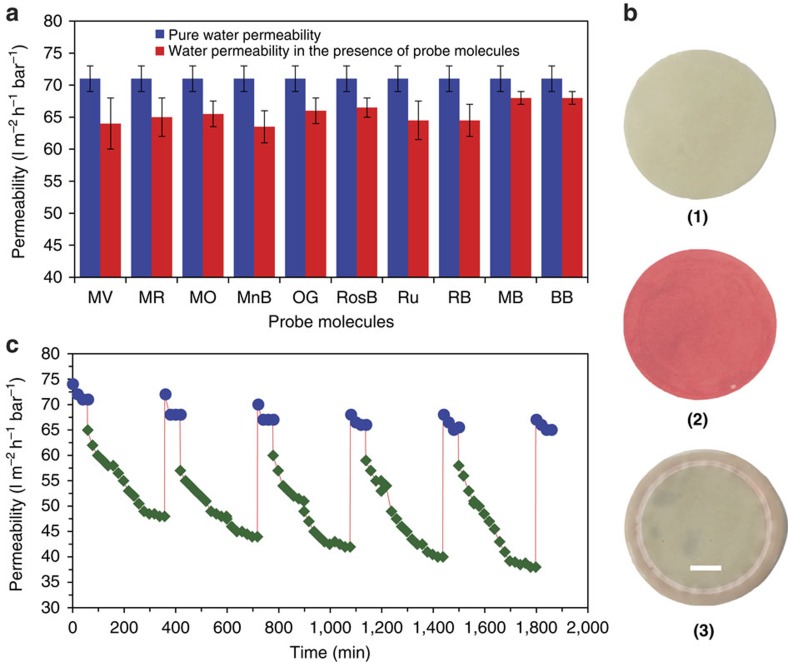
Flux regeneration in SAM. (**a**) Permeability declined during the filtration tests with probe molecules. The results show that a maximum of 10% decline is observed, it is larger for small molecules (methyl viologen ∼10% reduction), and less for bigger probe molecules (∼4.2% for methyl blue and brilliant blue) consistent with minimal sorption effects. Error bars are from five measurements showing the maximum and minimum values. (**b**) Photographs of the membrane before (**1**) and after filtration (**2**) of methyl red, and after the cleaning process (**3**) which shows regeneration of the parent-membrane surface (scale bar, 1 cm). (**c**) Demonstration of long-term viability, low-fouling behaviour of the membranes during filtration of BSA and flux recovery after chemical cleaning in five cycles. Each cycle commences with RO water permeation (Blue symbols), followed by permeation of BSA (Green symbols). Error bars in these figures are from five measurements showing the maximum and minimum values.

We evaluated the retention of this membrane for different probe molecules with different charges and hydrated radii; Methyl Viologen (positive charge, at pH 6), Methyl Orange (negative charge at pH 6), Methylene Blue (positive charge at pH 6.5), Orange G (negative charge at pH 6), Rhodamine B (electroneutral at pH 6 (electroneutral at pH 6), Tris(bipyridine) ruthenium(II) chloride (Ruthenium II) (positive charge at pH 6), Methyl Blue (negative charge at pH 6), Brilliant Blue (negative charge at pH 6.5) and Rose Bengal (negative charge at pH 6) (Fig. 5b). Before every experiment, the membranes are cleaned with ethanol, acetone and RO water followed by permeation of RO water until a stable permeability is observed. It is noteworthy that the cleaning process removed most of the probe molecules adhering to the membrane surface and almost 100% recovery of flux (Fig. 6a,b) is observed. Retention mechanism in membranes are reliant on size sieving, electrostatic repulsion and adsorption^41^,^42^, usually acting in tandem to affect separations. Sorption may dominate separations based on graphene-based materials^43^, so it is necessary to identify which of these mechanisms are crucial in our membrane. To calculate observation retention, *R*, (equation (2)) and the percentage of adsorption (equation (3)), we measured the concentration of each probe molecule in the feed (*C*_f_), the permeate (*C*_p_) and the retentate (*C*_r_)^44^ evaluated by measuring the absorbance of the relevant peaks using a ultraviolet–visible spectrometer.


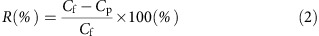



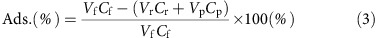


The membrane showed high retention (>90%) for the charged and uncharged solutes with a hydrated radius above 5 Å (Fig. 5b). Fig. 5c reports the analysis of feed, retentate and permeate concentration along with percentage retention and percentage adsorption in all the experiments. The results show that the retentate concentration is always larger than the feed concentration, while the adsorption percentage is <10%, irrespective of the probe molecule species in consideration. The permeability during filtration of the probe molecule was usually 90–95% of their clean water permeability (Fig. 6a) further supporting minimum sorption. Based on these measurements, one can argue that SAMs primarily sieve molecules when the average interlayer space of the graphene sheets approaches the physical size of the probe molecules, reported here as hydrated radius. It is also worth noting that the negatively charged probe molecules have higher retention than the positively charged molecules suggesting that electrostatic effects are also important.

### Salt retention

We evaluated the retention of monovalent and divalent salts (Na_2_SO_4_, MgSO_4_, MgCl_2_ and NaCl) at a concentration of 2 g l^−1^. The membrane showed retention between 30–40% for all the salts (Fig. 5d). The salt retention capability of the membrane is not surprising as the interlayer spacing is small (∼9.5 Å) and the membrane is abundant with various negatively charged oxygen functional groups, such as carboxyl, hydroxyl and epoxy, which persist even after the mild reduction used in stabilizing the membrane (Supplementary Fig. 4). These negatively charged groups particularly carboxylic acids, based on Donnan exclusion theory, will repel co-ions, and consequently retain counter ions to keep electroneutrality of the solution on each side of the membrane.

### Long-term filtration tests

A key attribute of our membrane is the stability in aqueous environments and that the retention is affected by sieving on the top surface of the membrane (Fig. 6b). This allows the membrane to be cleaned in polar and non-polar solvents for multiple reuse. Long-term filtration tests (over 24 h at 0.5 bar pressure) were carried out with BSA, a common laboratory model foulant in membrane-fouling studies^45^,^46^,^47^. The SAM showed fouling resistance and flux was recovered by a simple solvent cleaning (Fig. 6c). The fouling behaviour of membrane strongly depends on physical and chemical characteristics of the membrane surface such as pore size, porosity, pore morphology and most importantly the hydrophobicity^48^,^49^. Fortunately, our membrane retain hydrophilic groups (Supplementary Fig. 4) that decreases hydrophobic interaction with the organic probes and proteins^47^,^48^. As a result, a simple cleaning procedure using ethanol, acetone and RO water effectively recovered more than 90% of the flux after every cleaning cycle—this was true for the probe molecules and also for stronger foulant such as BSA (Fig. 6a–c).

### Homogeneity of large-area membrane

To demonstrate the homogeneity of the large-area membrane, four pieces were incised from a single large-area membrane and their performance was evaluated. (Supplementary Fig. 5). RO water permeability and retention of each individual membrane is shown in Supplementary Table 3. It is seen that each of these membranes have almost similar performance in water permeability (mean—76.25 l m^−2^ hr^−1^ bar^−1^ and s.d. of 5.7) and Methyl Red rejection (mean—91% and s.d. of 3) using methodology reported in the manuscript. These numbers are excellent evidence for the homogeneity of the large-area membrane.

## Discussion

The underlying principles of fluid-physics necessary for fabricating uniform and continuous graphene-based membranes by shear-induced alignment of liquid crystals of GO and the role of stacking order in enhancing water permeability is now discussed. The uniformity and continuity of the membrane arises from a competition between two factors: the casting of a uniform liquid film and then maintaining the stability of the liquid film during drying. The GO dispersions are shear-thinning, pseudoplastic fluids^27^,^50^ especially in high volume fractions and are highly viscous in zero-shear and very thin at high shear rate (Fig. 2b,c)—this is instrumental in obtaining a uniform membrane by shear alignment. For example, a GO dispersion at 40 mg ml^−1^ would have a zero-shear viscosity of 66 Pa s, but at a shear rate of 10^4^ s^−1^, relevant to our process, it will decrease to 0.0164 Pa s meaning that the nematic phase becomes fluid when forced under the micron scale outlet of the blade; membrane formation is also accentuated by the smaller surface tension and smaller contact angle of the nematic fluid (Table 1) to wet the underlying porous membrane. To obtain a uniform membrane it is critical to ensure that the processed liquid film from the GO dispersions remains uniform and continuous until it dries. If for any reason, the liquid film moves or migrates on the substrate, dewetting may ensue and the uniformity and continuity of the film degrades^26^,^51^,^52^ (Figs 2f and 3). Then to maintain stability of the liquid film during drying, the film needs to resist dewetting. In general, dewetting occurs on nonwettable substrates and can also be initiated by various film-thinning mechanisms, which persist until holes are produced and the film is ruptured. A large number of factors influence dewetting, such as solvent evaporation (especially in the case of low concentration dispersions), electrostatic repulsion (or attraction) forces between the dispersion and the substrate, dispersion migration due to gravity or capillary-driven flow, film thickness and viscosity and surface tension gradients. Among all film-thinning mechanisms, the predominant factor for dewetting is low viscosity and high surface tension of the dispersion^51^,^53^. The dewetting time can be estimated by 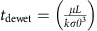
34,54, where *t*_dewet_ is the dewetting time (s), *σ*(N m^−1^) and *μ* (Pa s) are the surface tension and the viscosity of the dispersion, respectively, and *θ* (rad) is the contact angle between the dispersion and the substrate. *k* is a constant related to the fluid property and is assumed to be 10^−3^ for water-based system^34^,^54^. *L* is the length scale, which is estimated as 10% of the substrate width^34^. Drying time is the time between casting the liquid film and its solidification, which is defined by 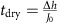, where Δ*h* is a parameter estimated to 80% of the thickness of the liquid film^34^ and *J*_0_ is the solvent evaporation current (cm s^−1^). To avoid rupture and obtain a continuous and uniform film, the drying time must be lower than the dewetting time. *J*_0_ was calculated by recording the mass loss of the liquid film on drying. The volume of the liquid film was calculated by considering the density of GO (∼1.8 g ml^−1^)^55^, using the mass and concentration of the GO dispersion. Dividing the volume with the area of the liquid film, we obtained the thickness of the liquid film during drying and subsequently calculated *J*_0_ (2 × 10^−6^ cm s^−1^). With increasing GO concentration, because of the enhanced zero-shear viscosity, lower surface tension and increase in the contact angle, the dewetting time-scale of the nematic fluids can be easily increased by over six orders of magnitude (Table 1). In fact, the dewetting time increased from 0.012 to 4,313 s with an increase in GO concentration from 0.1 to 40 mg ml^−1^ (Table 1). Since the drying time (40 s) in case of 40 mg ml^−1^ GO dispersion was significantly lower than the dewetting time (4313, s), uniform films could be produced under these conditions. Optical images and SEM in Fig. 3 confirm that dewetting in the films formed from such high concentrations is prohibited.

**Figure 7:**
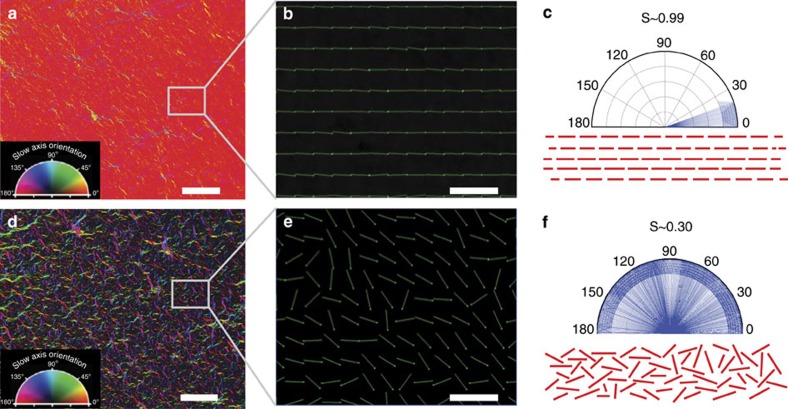
Effect of stacking of GO sheets on performance of filtration membranes. Polarized light imaging results of (**a**–**c**) SAM and (**d**–**f**) vacuum filtration membrane. (**a**,**d**) Falsely coloured polarized light images, where the hue represents the azimuth as depicted by the legend (scale bars are 50 μm). Regions with the same hue represent the same azimuth angle, so the SAMs have higher in-plane stacking order while vacuum filtration membranes have lower stacking order. This is supported by their slow axis vector representations (**b**,**e**), which are expanded view of the boxed areas (scale bar, 10 μm), polar histograms of the azimuth angles and the in-plane order parameters (**c**,**f**). (**c**,**f**) Predicted organization of graphene sheets in membranes.

**Figure 8:**
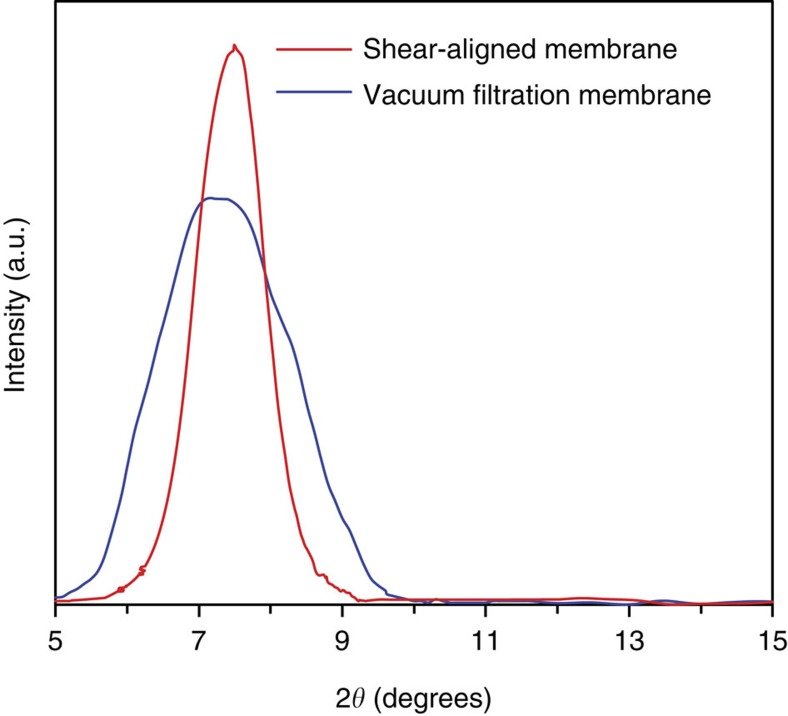
X-ray diffraction charactrization of SAM and vacuum filtration membrane. X-ray diffraction patterns of SAM and vacuum filtration membranes demonstrate highly ordered lamellar structure for SAM.

To elucidate the role of stacking order of graphene sheets, SAM and the vacuum filtration membrane are contrasted. Two independent techniques, polarized light imaging (Fig. 7a–f) and X-ray diffraction (Fig. 8) were used to confirm the ordering of the GO sheets. Polarized light imaging measures the local orientation order by imaging the slow axis of alignment of the graphene sheets in the plane of the membrane, while X-ray diffraction measures the crystalline order of the interlayer spacing. Polarized light imaging technique has been widely used for order parameter characterization of molecules and particles^56^,^57^ as well as GO sheets in liquids^16^,^18^,^58^ or solid state films^33^. We have compared the optical anisotropy of SAM with those synthesized by vacuum filtration^2^,^12^,^13^,^14^. Figs 7a,d show the processed false colour images of the azimuth orientation of the GO assemblies for SAM and vacuum filtration membranes where the hue represents the azimuth angle^33^. Similarly, Figs 7b,e display the vector representation of the azimuth angle. In comparison with the vacuum filtration membrane, a distinctly uniform hue (also represented as orientated azimuth vectors) was observed for SAM, suggesting that they exhibit a higher orientation order. We have used a scalar parameter, *S*, for the distribution of the azimuth angles in the *x*–*y* plane to quantify the alignment of the GO sheets. The scalar parameter is defined by 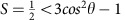
> (ref. 59), where *θ* is the angle between the mean azimuth (the director axis) and the azimuth at each pixel (the long axis of each graphene sheet). *S*=1 represents parallel alignment with the director and a perfectly oriented system, whereas *S*=0 represents a system with completely random orientations. SAM exhibit *S* values of ∼0.99, whereas vacuum-filtered membranes showed ∼0.3, demonstrating the high degree of in-plane order for SAM.

In addition, the X-ray diffraction patterns revealed that the broad range of interlayer space of GO sheets in vacuum filtration membrane contrasted with a narrow range for SAM (Fig. 8). This X-ray diffraction data is in good agreement with the results obtained by LC-PolScope (LPS) imaging and supports the conclusion that the lamellar structure of SAM are more organized and ordered than those obtained from vacuum filtration (Fig. 7a–f). Interestingly, the graphene sheets that comprise the film have a lateral dimension of ∼900 nm, while the membrane itself is much thinner (Fig. 2g and i)—this strongly suggests that the shear stress orients the sheets into the plane of the substrate. The in-plane orientation of the graphene sheets is consistent with theories of flow alignment of discotic nematic liquid crystals under a shear flow field^17^,^36^. This remarkably high order is unique to our processing approach and the key distinction of our shear-alignment method with other processing approach^2^,^12^,^13^,^14^ (Figs 7 and 8). We believe that the enhanced performance of our membrane is a consequence of the highly ordered graphene channels in the membrane plane. These highly ordered graphene sheets make well-organized and precise channels in the plane of the membrane that facilitate water transport^60^. In disordered membranes, the graphene sheets have random orientation that leads to disordered channels with broad range of sizes (Fig. 8). The random orientation of the graphene sheets could introduce multiple effects, such as, increased tortuosity, mechanical roughness and chaotic interconnectivity between GO sheets, which as a result increase the flow resistance of a membrane.

In summary, we have utilized the flow properties of a nematic GO fluid in developing a rapid and scalable process to produce large-area, thin, uniform and continuous graphene-based membranes supported on porous substrates. The shear-alignment process introduces large in-plane order and stacking periodicity in the graphene-based films. This structural order is found to enhance water flux dramatically while facilitating retention of organic molecules and ions by molecular sieving and electrostatic repulsion. The large-area graphene-based membranes produced by shear alignment have higher flux than commercially available Dow Filmtec NF270 membranes and excellent flux recovery by simple solvent cleaning. These membranes are ideal candidates for highly desirable low-pressure, low-fouling, membrane-based nanofiltration separations.

## Methods

### Synthesis of GO dispersions

GO was synthesized using modified Hummers' method^61^. SP-1 grade 325 mesh graphite powder (Bay Carbon, Inc.), sulfuric acid, potassium persulfate, phosphorus pentoxide and potassium permanganate (Sigma-Aldrich), were used for the synthesis. The synthesized GO was exfoliated by sonication (UP-100 Ultrasonic processor) in RO water for 1 h, followed by centrifugation to remove the un-exfoliated GO. The average lateral size of the GO sheets was determined using a SEM (FEI Nova NanoSEM 450 FEGSEM (2012)) and was estimated to be ∼0.9±0.4 μm (90 sheets were measured to calculate the average sheet size). An Ocean Optics USB4000 ultraviolet–visible spectrometer was used to determine the GO concentrations by measuring the absorbance at 230 nm (using a quartz cuvette, Starna Cells Pty. Ltd, Australia). Various concentrations of GO dispersions were prepared using a superabsorbent polymer (cross-linked polyacrylate copolymer based hydrogel beads, Demi Co., Ltd, China). For example, within ∼1 h, a 10 ml GO dispersion with a concentration of 20 mg ml^−1^ was obtained from a 1 l suspension of 0.25 mg ml^−1^ GO using 10 g of the hydrogel beads. To avoid possible concentration polarization around the beads and to speed up the absorbent process the container was mildly agitated by a magnetic stirrer. After the hydrogel beads were saturated with water, they were removed from the concentrated solution. The GO deposits adhering to the surface of the saturated beads were removed by washing them with RO water. The saturated hydrogel beads could be reused after drying them at 50 °C overnight.

### Raman spectroscopy

The GO sample was prepared by drop casting of GO (5 mg ml^−1^) on glass slide followed by drying overnight under ambient lab conditions. The saturated hydrogel beads (swollen in either 5 mg ml^−1^ of GO or in pure RO water) were cut by a stainless steel scalpel blade and mounted onto a glass slide. Raman spectra of GO and of the swollen hydrogel beads were obtained using a Renishaw Confocal micro-Raman Spectrometer equipped with a HeNe (632.8 nm) laser operating at 10% power. Extended scans (10 s) were performed between 100 and 3,200 wave numbers with a laser spot size of 1 μm. Once the background was removed, the intensity of the spectra was normalized by dividing the data with the maximum intensity. The peak positions were found using the full-width at half-maximum, as is common practice for analysing spectral data.

### Rheological measurements

A HAAKE MARS II Rheometer (Thermo Electron Corporation, Germany) was used to measure the viscosity of GO dispersions. A titanium coated cone-plate with a 60 mm diameter and a cone angle of 1° was used. The temperature inside the cone-plate was fixed to 22.00±0.01 °C by using a Peltier system and a thermostat HAAKE Phoenix II (Thermo Electron Corporation). The experiments were performed using 2 ml dispersions with a constant gap of 0.041 mm. We evaluated zero-shear viscosity by measuring the viscosity of the GO dispersions at a shear rate of 0.001 s^−1^ (ref. 24).

### Surface tension and contact angle measurements

A custom-designed pendent drop apparatus^62^ was used to measure the surface tension of the GO dispersions as a function of the concentration. A drop was formed at the end of a capillary with 0.7 mm diameter, and a digital CMOS camera monitored the shape of the droplet. A customized software^62^ determined the surface tension of these dispersions by comparing the actual curvature with the theoretically predicted curvature of the droplet estimated by Young–Laplace correlation^62^. The static contact angles between the GO dispersions and the Nylon substrates were measured by placing a droplet of a GO dispersion (∼3 μl of volume) on a Nylon substrate using a capillary with a diameter of 0.7 mm. The digital camera was used to monitor the shape of the droplet immediately after the droplet deposition. The average value of the contact angle was determined from the measurements of the contact angles at five different locations on a Nylon substrate.

### Lab-scale membrane fabrication by doctor blade

To evaluate film stability and dewetting phenomena and obtain the optimal conditions required for production of a continuous GO film, a simple lab-scale doctor blade (MTI Corporation, USA) was used for various GO concentrations. The doctor blade has a rectangular outlet formed between the blade and the substrate, through which the movable blade spreads the GO dispersion on the substrate (Fig. 1d). The doctor blade gap size was ∼1 μm and the casting speed was ∼1 cm s^−1^. Typically, to prepare a GO film, 1 ml of a GO dispersion was spread over a porous Nylon substrate (Nylon 66 membrane, pore size 0.2 μm, 5 × 5 cm^2^, MDI, India) using the doctor blade. A syringe pump was used to precisely control the movement of the doctor blade. Subsequently, the resultant liquid films were dried overnight under ambient conditions.

### Semi-industrial scale membrane fabrication

Large-area, continuous, supported GO membranes were prepared using a conventional gravure printing machine (Labratester, Norbert Schläfli Machinery Company, Switzerland; Fig. 2h). To produce GO membranes, small quantities of GO dispersion were placed on the printing plate, which was spread by a doctor blade. Subsequently, a rubber-coated roller pressed the substrate on to the printing plate and transferred the liquid film from the printing plate to the substrate (Supplementary Movie 1). GO membranes were prepared using GO dispersions (40 mg ml^−1^) and 13 × 14 cm^2^ porous Nylon substrates (Nylon 66, pore size 0.2 μm, MDI, India). GO membranes with different thicknesses were prepared by repeating the printing process several times on the same substrate.

### Membrane fabrication by vacuum filtration

We used a 10 μg l^−1^ GO solution (from same stock of GO) and filtered it through the same porous Nylon support (Nylon 66, pore size 0.2 μm, MDI, India) using a vacuum filtration pump (KNF pump, model: N 810(3) FT.18). Different thicknesses of membrane were made by changing the volume of the GO solution.

### Polarized light imaging

Microscopy was carried out using a Leica DM IRB microscope with a LPS Abrio imaging system from CRI, Inc. (ref. 33) LPS imaging required GO membranes to be transferred onto glass slides. Nylon substrates supporting the GO membranes were etched using concentrated hydrochloric acid and the obtained free-standing GO membrane was transferred to a microscope glass slide. The order parameter, *S*, was calculated from the azimuth data (1,000 pixels).

### X-ray diffraction

X-ray diffraction patterns of the GO membranes were obtained using a Phillips 1140 diffractometer with Cu Kα line generated at 40 kV and 25 mA at a scan rate of 1° min^−1^, and a step size of 0.02°. X-ray diffraction samples were prepared by etching the Nylon substrates with concentrated hydrochloric acid (Sigma-Aldrich) and by transferring the free-standing GO films on glass slides.

### Scanning electron microscopy

The uniformity and continuity of these GO films were then analyzed by a high resolution SEM (FEI Nova NanoSEM 450 FEGSEM (2012)), typically operating at 5 keV. All the samples were coated with Iridium by a Cressington 208 HR sputter coater. For cross-sectional imaging, the Nylon supported GO films were cut into rectangular strips, which were soaked in liquid nitrogen for 30 s and were then carefully snapped with flat tweezers. Cross-sections were mounted vertically on a metal stub and imaged at 15 keV.

### Atomic force microscopy

Free-standing GO membranes were prepared by etching nylon substrates in concentrated hydrochloric acid (Sigma-Aldrich). Subsequently, these free-standing GO films were transferred to microscope glass slides. Atomic force microscopy measurements were carried out using a JPK Nanowizard 3 to calculate the thickness of GO membranes. This instrument is equipped with capacitive sensors to ensure accurate reporting of height, *z* and *x*–*y* lateral distances. Imaging was performed in tapping mode using a Bruker NCHV model cantilevers with diameter 10 nm, with nominal resonant frequencies of 340 Hz, spring constant of 20−80 N m^−1^. Images were obtained with a set-point force of 1 nN. The cantilever drive frequency was chosen to be 5% smaller than the resonance frequency. The thickness of the GO films were estimated from the height difference between the glass and the GO films from three different positions using a line scan as is shown in Fig. 2i for the membrane with a thickness of 150±15 nm.

### Fourier transform infrared spectroscopy

To evaluate the presence of functional groups in the GO membrane and partially reduced membrane, FTIR spectra of the membranes were recorded using an attenuated total reflectance Fourier transform infrared (PerkinElmer, USA) in the range of 500−4,000 cm^−1^ at an average of 32 scans with a resolution of 4 cm^−1^.

### Nanofiltration characterization of the membranes

The SAMs were cut into the required size (47 mm diameter) for filtrations tests. To increase the water stability of the SAMs, they were partially reduced by exposing to 0.02 ml of hydrazine hydrate vapour (88%, Merck) for 5 min by placing a hermetically sealed vessel containing the GO membrane onto a hot-plate at 60 °C. The water permeability and retention capabilities of the membranes were examined using a commercial bench-scale stainless steel dead-end stirred cell-filtration unit (Sterlitech HP4750; Supplementary Fig. 6). The effective membrane area was ∼13.6 cm^2^ and all the experiments were performed at ambient conditions (∼21 °C) with a nitrogen pressure of 0.5 bar. Permeability of the membrane (with units 1 h^−1^ m^−2^ bar^−1^) for pure water or water/probe molecule solutions was determined after a constant flux was obtained, typically after 1 h of permeation, and calculated by:


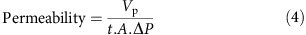


Where *V*_p_ is the permeate volume, *t* is the permeation time, *A* is the active area of the membrane and Δ*P* is the imposed nitrogen pressure. To evaluate the retention performance of the membrane the stirred cell was filled with 10 mg l^−1^ of the test solutions. To diminish the role of adsorption, the membrane were pre-saturated by filtering ∼20 ml of the test solution and then, to remove any solute adhered to the membrane surfaces, the membranes were thoroughly washed with ethanol, acetone and finally RO water (typically 50 ml of the solvent was added to the filtration cell and left stirring at 800 r.p.m. for 5 min). The retention performance of the membranes were evaluated by filling the cell with 100 ml of solution followed by applying a pressure to the membrane and allowing 20 ml to permeate through it. The 20 ml, which permeated through the membrane, and the 80 ml retentate were both collected and analysed. All tests were repeated five times. For accurate estimation of the concentration of the probe molecule in the retentate stream, we rinsed all the components which were in contact with the retentate solution in the filtration cell, such as, the stirring apparatuses, interior walls of the cell and the top surface of the membranes with 100 ml of RO water and accounted for during calculation of the retentate concentration.

### Salt retention performance

The salt retention performance of the GO membrane was examined by evaluating the retention of the selected monovalent and divalent salts, such as, Na_2_SO_4_, MgSO_4_, MgCl_2_ and NaCl, with a concentration of 2 g l^−1^. The filtration tests were performed using the same dead-end cell, with a nitrogen pressure of 0.5 bar. To minimize the concentration polarization effect on the retention performance, the feed solution was stirred at 800 r.p.m. during the filtration. The tests were started by recording the permeability of the membranes for RO water until a stable condition was achieved (typically after 1 h). Subsequently, RO water is replaced by 50 ml of the salt solution. The salt retention performance of the membranes were evaluated by filtering 10 ml of the initial feed. Before every experiment, the membrane was cleaned by filtering RO water through them until the permeability became stable and also evidence of salts were observed (typically after 1 h) in the permeate. The retention performances of the membrane for the salts were calculated using the equation (2). The concentrations of the salts were measured by an ion conductivity metre (TPS Aqua C, Thermo Fisher Scientific).

### Long-term viability and membrane reuse

To evaluate the long-term viability and the reusability of our membranes, we have filtered 100 p.p.m. of BSA. The fouling tests were performed in the dead-end stirred filtration cell (Sterlitech HP4750) attached with a 4.5 l dispensing vessel, under constant stirring at 800 r.p.m. (to minimize concentration polarization) and a nitrogen pressure of 0.5 bar. The test was started by recording permeability of the membrane for RO water until a constant flux was obtained, typically after 1 h (*j*_w,1_). The initial RO feed was removed and replaced by the BSA solution. On the commencement of filtration with 0.5 bar pressure, permeate was weighed and collected using a Sartorius scale customized with a Labview interface. This completes the first cycle. Once the BSA test was completed, the membrane was cleaned by ethanol, acetone and RO water. The filtration of the BSA solution was continued for 5 h (five cycles). The aforementioned procedure is now repeated again with the second RO water permeability designated as *j*_w,2_. Five cycles were completed in total (Fig. 6c). Although we understand that industrially relevant cleaning protocols for membranes require the use of alkalis and acids, the choice of the cleaning solution for our studies was based on the ability of the GO membranes to have chemical resistance towards solvents such as acetone and ethanol.

Antifouling behaviour of the SAM due to the chemical cleaning was evaluated by the flux recovery (FR), which is calculated by the following equation:


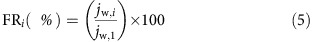


Where, *j*_w,1_ is the initial flux of the membrane for RO water before the first cycle, *j*_w,*i*_ is the membrane flux (after cleaning the membrane by ethanol and acetone and RO water) for RO water after cycle *i*.

### Estimation of hydrated radius of probe molecules

The hydrated radii of most probe molecules used in this study are not well-known with the exception for Ruthenium II^2^,^63^, but the hydration radius is relevant to interpreting our nanofiltration studies. Various studies have estimated the hydrated radii of different molecules by using a correlation between more easily obtainable size parameters such as Stoke's radius^2^,^64^ or crystal radius^64^, and physical parameters such as viscosity^64^,^65^,^66^ with hydration radius. Here we have estimated the Connolly accessible area (CAA) calculated by Chem3D software, which is similar to the method of Van der Bruggen *et al.*^67^ that utilized an energy minimization routine to estimate the molecular size of probes used in nanofiltration studies. The CAA is described by the locus of the centre of the solvent molecule (which is considered as a sphere) as it is rolled around the probe molecules van der Waals surface^68^. We chose several molecules (in the molecular weight range of our probe molecules) with known hydrated radii to perform the CAA calculation. To calculate the CAA, the structure of the molecules were generated using Chem3D Pro 13.0 (Cambridge-Soft, MA, USA) and the energy minimization was performed by molecular mechanics calculation, using the MM2 method. Once the CAA was determined we calculated the equivalent spherical radius (CAA radius), these values are listed in Supplementary Table 4. Supplementary Fig. 7 shows the correlation between the obtained CAA radius and hydrated radii obtained using this method. We used this correlation to estimate hydrated radius of probe molecules whose hydration radius was unknown.

## Additional information

**How to cite this article:** Akbari, A. *et al.* Large-area graphene-based nanofiltration membranes by shear alignment of discotic nematic liquid crystals of graphene oxide. *Nat. Commun.* 7:10891 doi: 10.1038/ncomms10891 (2016).

## Supplementary Material

Supplementary Information Supplementary Figures 1-7, Supplementary Tables 1-4, Supplementary Notes 1-2 and Supplementary References.

Supplementary Movie 1 Large-scale production of graphene based membrane

## Figures and Tables

**Table 1:** Physical properties of GO dispersion.

**Concentration (mg ml**^**−1**^**)**	**Volume fraction**	**Surface tension (mN m**^**−1**^**)**	**Contact angle (°)**	**Zero-shear viscosity (at 10**^**−3**^** s**^**−1**^**) (Pa s)**	**Apparent viscosity (at** **10**^**4**^** s**^**−1**^**) (Pa s)**	**Dewetting time (s)**	**Drying time (s)**
0.1	0.005	71.9	81	0.00128	0.0016	0.012	40
5	0.27	68	70	0.0094	0.002	0.151	40
10	0.55	66	67	0.8	0.0041	15	40
40	2.22	49	49	66	0.0164	4313	40

Physical properties of a typical isotropic (0.1 mg ml^−1^), onset of bi-phasic (5 mg ml^−1^), bi-phasic (10 mg ml^−1^) and fully nematic (40 mg ml^−1^) colloidal dispersions of graphene oxide (GO) demonstrating a decrease in surface tension and contact angle which promotes wetting of the fluid on the porous substrate and an increase in dewetting time compared with drying time.
