# Supplementary material for: Large-area graphene-based nanofiltration membranes by shear alignment of discotic nematic liquid crystals of graphene oxide
Source: Nat Commun. 2016 Mar 7;7:10891. doi: 10.1038/ncomms10891 (PMC4786680; doi:10.1038/ncomms10891)
Supplement: Supplementary Information — Supplementary Figures 1-7, Supplementary Tables 1-4, Supplementary Notes 1-2 and Supplementary References. [file ncomms10891-s1.pdf]

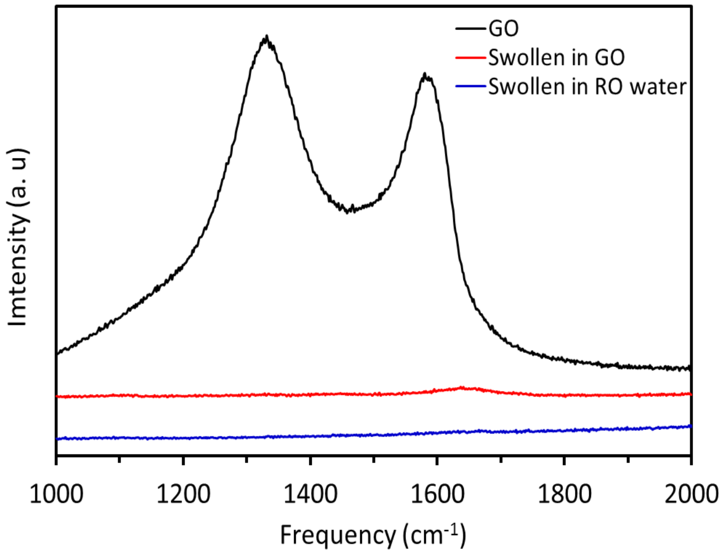

Supplementary Figure 1 Raman spectra of GO, the hydrogel bead swollen in GO solution and the hydrogel bead swollen in RO water.

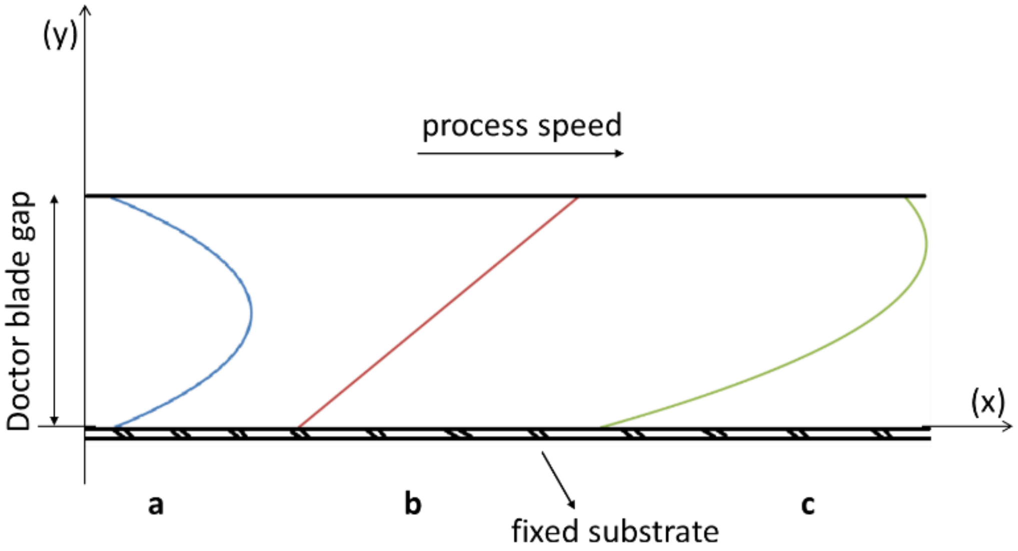

Supplementary Figure 2 Schematic of velocity profile in doctor blade channel for: (a) channel flow, (b) couette flow and (c) total flow.

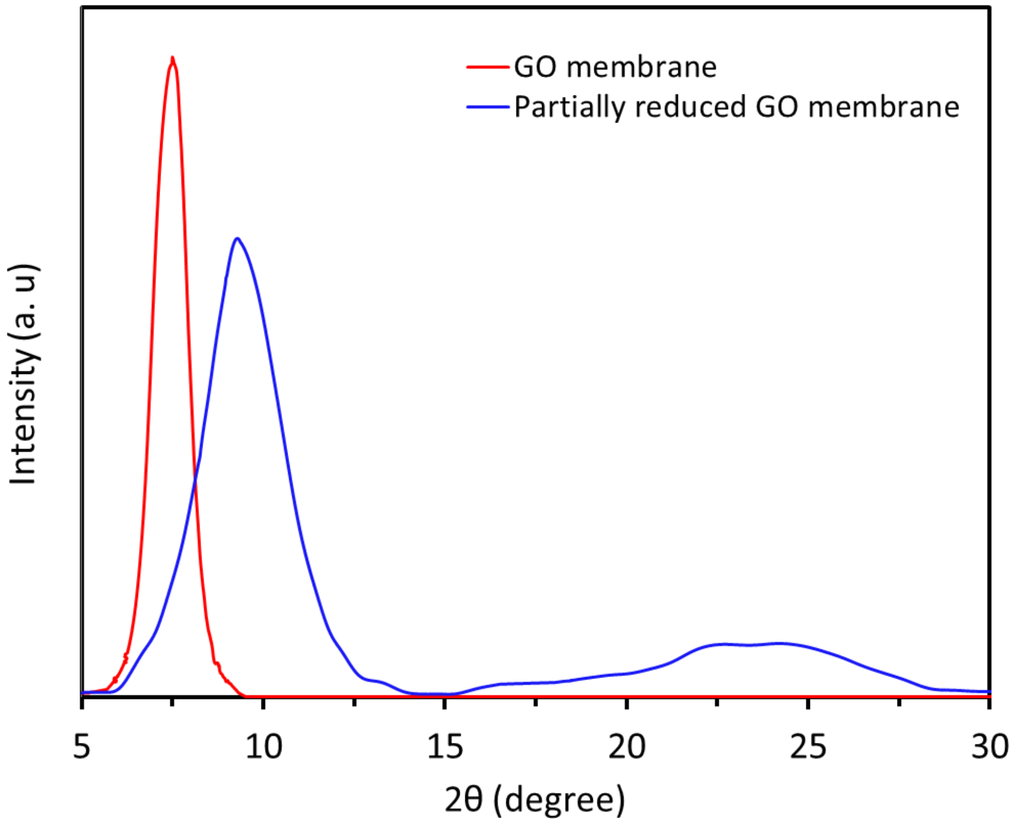

Supplementary Figure 3 The XRD patterns of the SAM of GO and SAM after partial reduction in hydrazine vapour. We measured the average interlayer space between GO sheets in the partially reduced SAM to estimate the pore size relevant to our filtration experiments. XRD of the partially reduced membrane showed an intense peak at 2θ=9.3°, which corresponds to an interlayer spacing of 9.5Å. The presence of a weak, broad peak between 2θ=15.4° and 2θ=28.6° indicates that the GO membrane has indeed been partially reduced.

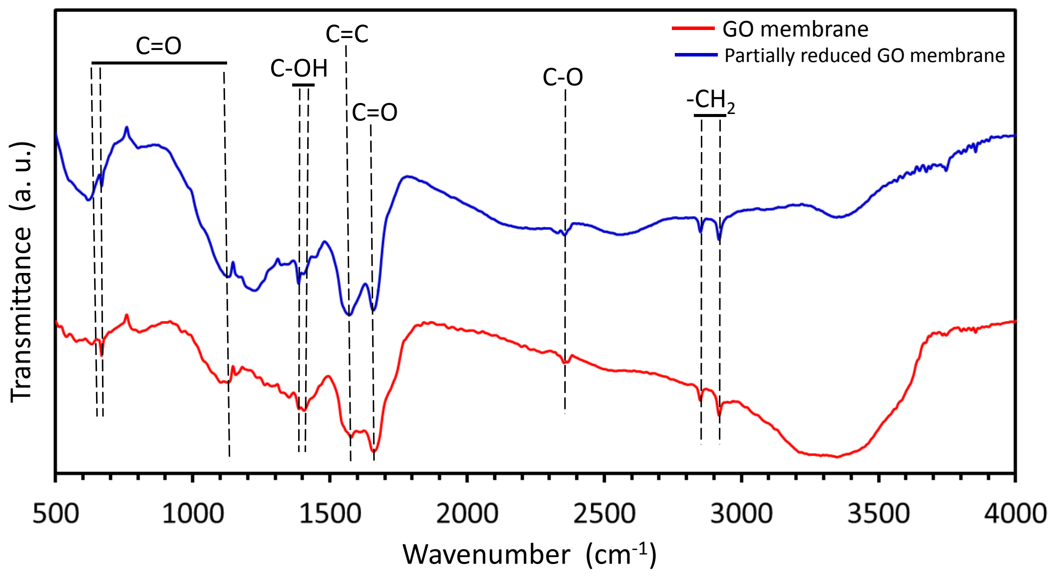

Supplementary Figure 4 The FTIR spectra of SAM of GO and SAM after partial reduction in hydrazine vapor. The overall ATR-FTIR spectra of GO and the partially reduced GO films both consisted of the typical GO oxygen functional groups such as C=O (~630 cm^{-1} – ~1150 cm^{-1} and ~1650 cm^{-1}), C-OH (~1380 cm^{-1}) and C-O (~2390 cm^{-1}). In case of the partially reduced GO film, the intensities of the peaks at 1650 cm^{-1} (corresponding to C=O) and ~ 3400 cm^{-1} (corresponding to C-OH) have significantly decreased, while the peak at 1570 cm^{-1} (corresponding to C=C) has significantly increased, confirming a partial reduction of the GO membrane, in agreement with the observed XRD spectra (Supplementary Fig. 3) for these two specimens.

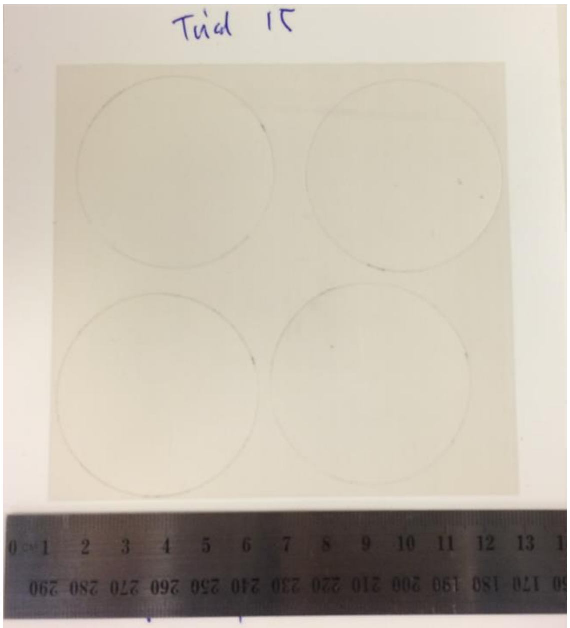

Supplementary Figure 5 Homogeneity of the large area shear-aligned membrane. Image demonstrate how we incised four membranes from one single piece of large-area membrane to examine homogeneity of the membrane.

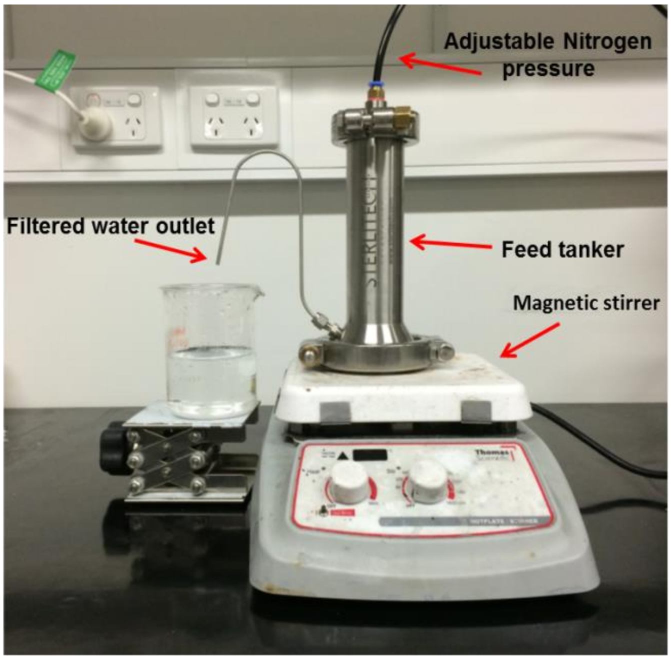

Supplementary Figure 6 A dead-end filtration cell (Sterlitech HP4750 with high pressure stirred cell) pressurized by nitrogen gas was used to examine the performance of the membranes.

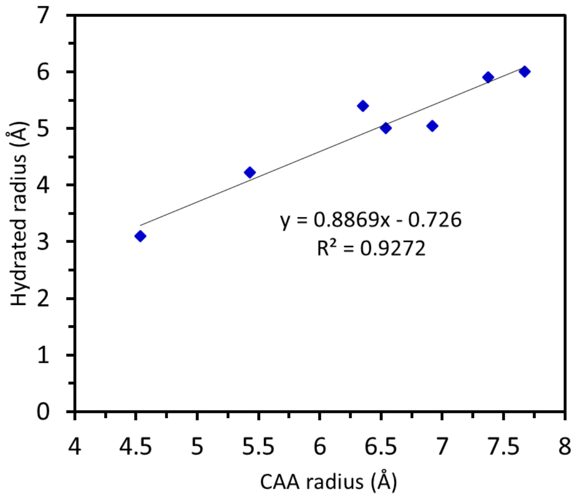

Supplementary Figure 7 Correlation between CAA radius and hydrated radius of several well established probe molecules. We used this correlation to estimate hydrated radius of our probe molecules.

Supplementary Table 1 Extrapolation of the surface tension to estimate surface tension at 40 and 60 mg ml^{-1} concentration.

**GO concentration (mg ml^{-1})**	**0**	**0.1**	**2.5**	**5**	**10**	**15**	**20**	**40**	**60**
**GO surface tension (mN m^{-1})**	72.5± 2.1	71.9 ± 2.5	69.6 ± 1.6	68.0 ± 1.9	66.0 ± 1.3	65.1 ± 2.8	60.0 ± 1.6	~49	~39

Supplementary Table 2 Extrapolation of contact angle to estimate contact angle at high GO concentration.

**GO concentration ($mg\ ml^{-1}$)**	**0**	**0.1**	**2.5**	**5**	**10**	**15**	**20**	**40**	**60**
**Contact angle (degree)**	83 ±6	81 ± 4	73 ± 2	70 ± 3	67 ± 4	64 ± 5	62 ± 3	~49	~36

Supplementary Table 3 Nanofiltration performance variation check among four different samples from a single large area membrane.

Sample (portion of sheet)	Water Permeance ($l\,h^{-1}m^{-2}bar^{-1}$)	Rejection
1 (top left)	76	91%
2 (top right)	75	94%
3 (bottom left)	70	93%
4 (bottom (right)	84	87%

Supplementary Table 4 List of hydrated radii of several known molecules and hydrated radii of our probe molecules, estimated by the correlation between the hydrated radius and the radius estimated from the Connolly Accessible Area (CAA). Radii which are not followed by references have been estimated.

**Molecule**	**Molecule structure**	**Connolly accessible area (Å^2)**	**Radius based on CAA (Å)**	**Hydrated radius (Å)**
Ruthenium II	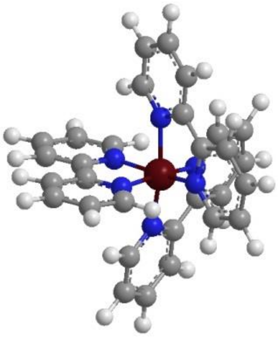	683.00	7.37	5.9 [1, 2]
Sucrose	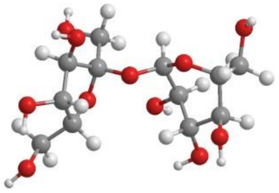	537.59	6.54	5.01 [2]
Ferricyanide ion	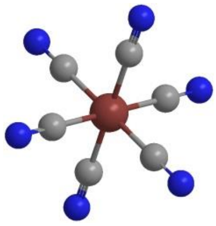	370.50	5.43	4.22 [2]
Raffinose	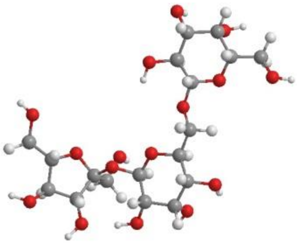	740.17	7.67	6 [3]

Lactose	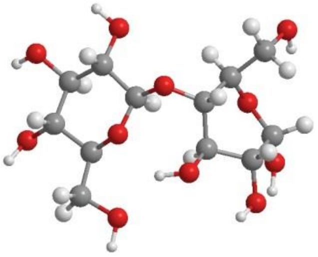	507.37	6.35	5.4 [3]
Pyrenetetrasulfonic acid ion (PTS^{-4})	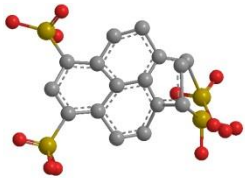	601.08	6.92	5.04 [2]
Glycerol	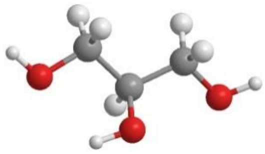	258.42	4.53	3.1 [3]
Methyl Viologen ion	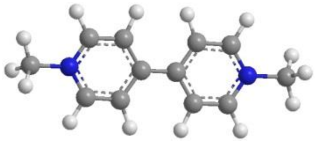	413.14	5.73	4.36
Methyl Red	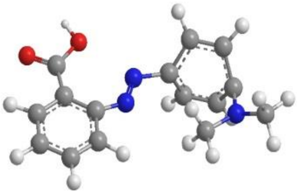	501.10	6.32	4.87
Methyl Orange ion	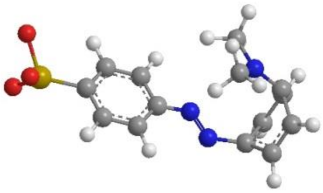	515.87	6.407	4.96
Methylene Blue ion	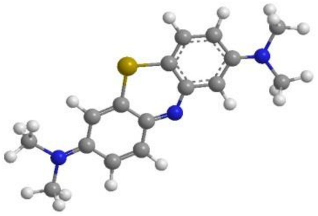	530.26	6.46	5.04

Orange G ion	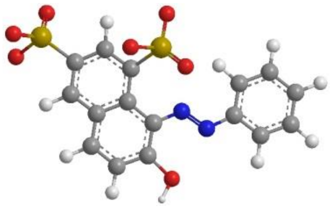	563.01	6.69	5.21
Rhodamine B	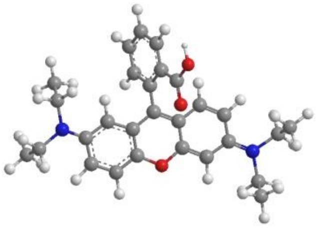	757.02	7.76	6.158
Methyl Blue ion	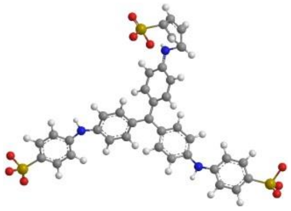	1026.64	9.04	7.29
Brilliant Blue G ion	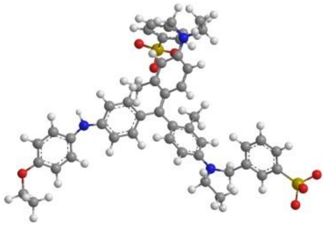	1211.60	9.82	7.98
Rose Bengal ion	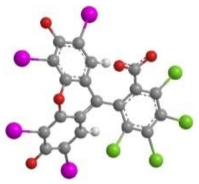	697.21	7.45	5.88

Supplementary Note 1:

Hydrogel bead Raman spectroscopy characterization

Raman spectroscopy was used to investigate the possibility that GO is absorbed into the hydrogel beads along with water. GO has two characteristic Raman peak at ~1330 cm^{-1} (D band) and ~1580 cm^{-1} (G band) corresponding to the amount of defects and in-plane bond stretching of sp^2 carbon atoms respectively. If the hydrogel beads have absorbed GO during the concentration process, Raman spectra of the swollen beads would display these characteristic peaks. To this end, we have compared the Raman spectra of hydrogel beads which have swelled after soaking in a GO solution to two control spectra: a GO film and hydrogel beads which have swelled after soaking in RO water. Supplementary Figure 1 demonstrated swollen beads in GO or RO water have similar characteristics peak and there is no evidence of GO peaks in the bead swollen in GO.

Supplementary Note 2:

Effective physical parameters in the casting process

To elucidate the essential physical parameters in the doctor blading process, many researchers [4, 5, 6] have used the generic Navier-Stokes equations to analyse the flow dynamics of the casting liquid in a rectangular channel of doctor blade assuming that the fluid flow is Newtonian. The fluid flow in the doctor blade gap is governed by a combination of pressure driven flow (resulting from the hydrostatic pressure of the GO dispersion in front of the blade) and the Couette flow (resulting from the external shear of the blade) [5] (Supplementary Fig. 2). The velocity profile is calculated by:

$$u(y) = \frac{Uy}{h_0} + \frac{1}{2\mu}\frac{\rho g(y - H)}{L} y(y - h_0) \qquad \text{(S 1)}$$

Here, u is the flow velocity in the y direction, U is the casting process speed, μ and ρ are the apparent viscosity and the density of the GO dispersion, respectively. H is the height of the GO dispersion in front of the doctor blade, h_0 and L are the dimensions of the blade gap (Fig. 2d). Based on our experimental conditions (U=1 cm s^{-1}, h_0 =10^{-6} m), the external shear of the blade dominates the fluid behaviour in the blade gap, therefore the total velocity profile of the dispersion in the blade gap may be considered as:

$$u(y) = \frac{Uy}{h_0} \qquad \text{(S 2)}$$

Here, the shear rate is calculated as:

$$\mathring{\gamma} = \frac{du}{dy} = \frac{U}{h_0} \qquad \text{(S 3)}$$

and the shear stress is defined by: $\tau = \eta\mathring{\gamma}$, therefore:

$$\tau = \eta\frac{U}{h_0} \qquad \text{(S 4)}$$

Supplementary Equation 4 estimates the total shear stress that the doctor blade imparts on the GO dispersions. Since the casting velocity and the doctor blade gap were kept constant during the experiments, the shear stress is dependent on the viscosity of the GO dispersion. Thus the imposed high shear stress (Table 1) aligns the nematic phase GO sheets [7, 8].

Supplementary References

1. Fornasiero F, *et al.* Ion exclusion by sub-2-nm carbon nanotube pores. *Proceedings of the National Academy of Sciences* **105**, 17250-17255 (2008).

2. Joshi RK, *et al.* Precise and ultrafast molecular sieving through graphene oxide membranes. *Science* **343**, 752-754 (2014).

3. Schultz SG, Solomon A. Determination of the effective hydrodynamic radii of small molecules by viscometry. *The Journal of general physiology* **44**, 1189-1199 (1961).

4. Pitchumani R, Karbhari VM. Generalized fluid flow model for ceramic tape casting. *Journal of the American Ceramic Society* **78**, 2497-2503 (1995).

5. Chou YT, Ko YT, Yan MF. Fluid flow model for ceramic tape casting. *Journal of the American Ceramic Society* **70**, C-280-C-282 (1987).

6. Kim HJ, Krane MJM, Trumble KP, Bowman KJ. Analytical fluid flow models for tape casting. *Journal of the American Ceramic Society* **89**, 2769-2775 (2006).

7. Carlsson T. Remarks on the flow alignment of disc like nematics. *Journal de Physique* **44**, 909-911 (1983).

8. Carlsson T. The possibility of the existence of a positive Leslie viscosity α2. Proposed flow behavior of disk like nematic liquid crystals. *Molecular crystals and liquid crystals* **89**, 57-66 (1982).

9. Nightingale Jr E. Phenomenological theory of ion solvation. Effective radii of hydrated ions. *The Journal of Physical Chemistry* **63**, 1381-1387 (1959).

10. Kurucsev T, Sargeson A, West B. Size and Hydration of Inorganic Macroions from Viscosity and Density Measurements. *The Journal of Physical Chemistry* **61**, 1567-1569 (1957).

11. Van der Bruggen B, Schaep J, Wilms D, Vandecasteele C. Influence of molecular size, polarity and charge on the retention of organic molecules by nanofiltration. *Journal of Membrane Science* **156**, 29-41 (1999).

12. Connolly ML. Solvent-accessible surfaces of proteins and nucleic acids. *Science* **221**, 709-713 (1983).
